# The Achilles’ heel of prevention to mother-to-child transmission of HIV: Protocol implementation, uptake, and sustainability

**DOI:** 10.1080/17290376.2017.1375425

**Published:** 2017-09-19

**Authors:** Violeta J. Rodriguez, Richard P. LaCabe, C. Kyle Privette, K. Marie Douglass, Karl Peltzer, Gladys Matseke, Audrey Mathebula, Shandir Ramlagan, Sibusiso Sifunda, Guillermo “Willy” Prado, Viviana Horigian, Stephen M. Weiss, Deborah L. Jones

**Affiliations:** ^a^ MSEd is a Senior Research Associate at the Department of Psychiatry and Behavioral Sciences, University of Miami Miller School of Medicine, Miami, FL, USA; ^b^ BA, is a Volunteer Research Assistant at the Department of Psychiatry and Behavioral Sciences, University of Miami Miller School of Medicine, Miami, FL, USA; ^c^ is a senior undergraduate student in the Department of Biology and Research Assistant in the, Department of Psychiatry and Behavioral Sciences, University of Miami Miller School of Medicine, Miami, FL, USA; ^d^ BS, is a third-year medical student at the University of Miami Miller School of Medicine, Miami, FL, USA and pursuing joint Doctor of Medicine and Master of Public Health degrees; ^e^ PhD, is a distinguished research fellow in the HIV/AIDS/STIs and TB (HAST) Research Programme, Human Sciences Research Council, Pretoria, South Africa; ^f^ MPH, is a Senior Researcher/PHD research trainee in the HIV/AIDS/STIs and TB (HAST) Research Programme, Human Sciences Research Council, Pretoria, South Africa; ^g^ BA(Hons), is a Project Supervisor in the HIV/AIDS/STIs and TB (HAST) Research Programme, Human Sciences Research Council, Pretoria, South Africa; ^h^ MDevSt, is a Research Specialist in the HIV/AIDS/STIs and TB (HAST) Research Programme, Human Sciences Research Council, Pretoria, South Africa; ^i^ PhD, MPH, is Chief Research Specialist at the HIV/AIDS/STIs and TB (HAST) Research Programme, Human Sciences Research Council, Pretoria, South Africa; ^j^ PhD, is the Dean of the Graduate School, the Leonard M. Miller Professor of Public Health Sciences, Miami, FL, USA; ^k^ MD, is Associate Professor at the Department of Public Health Sciences, University of Miami, Miller School of Medicine, Miami, FL, USA; ^l^ MD, is a Professor at the Department of Psychiatry and Behavioral Sciences, University of Miami Miller School of Medicine, Miami, FL, USA; ^m^ is a Professor at the Department of Psychiatry and Behavioral Sciences, University of Miami Miller School of Medicine, Miami, FL, USA

**Keywords:** implementation science, PMTCT, HIV, South Africa, implementation research, VIH et le SIDA, prévention de la transmission mère-enfant (PMTE), Afrique du Sud

## Abstract

The Joint United Nations Programme on HIV and AIDS proposed to reduce the vertical transmission of HIV from ∼72,200 to ∼8300 newly infected children by 2015 in South Africa (SA). However, cultural, infrastructural, and socio-economic barriers hinder the implementation of the prevention of mother-to-child transmission (PMTCT) protocol, and research on potential solutions to address these barriers in rural areas is particularly limited. This study sought to identify challenges and solutions to the implementation, uptake, and sustainability of the PMTCT protocol in rural SA. Forty-eight qualitative interviews, 12 focus groups discussions (*n* = 75), and one two-day workshop (*n* = 32 participants) were conducted with district directors, clinic leaders, staff, and patients from 12 rural clinics. The delivery and uptake of the PMTCT protocol was evaluated using the Consolidated Framework for Implementation Research (CFIR); 15 themes associated with challenges and solutions emerged. Intervention characteristics themes included PMTCT training and HIV serostatus disclosure. Outer-setting themes included facility space, health record management, and staff shortage; inner-setting themes included supply use and availability, staff–patient relationship, and transportation and scheduling. Themes related to characteristics of individuals included staff relationships, initial antenatal care visit, adherence, and culture and stigma. Implementation process themes included patient education, test results delivery, and male involvement. Significant gaps in care were identified in rural areas. Information obtained from participants using the CFIR framework provided valuable insights into solutions to barriers to PMTCT implementation. Continuously assessing and correcting PMTCT protocol implementation, uptake and sustainability appear merited to maximize HIV prevention.

## Résumé

Le programme conjoint des Nations Unies sur le VIH et le SIDA avait proposé de réduire les chiffres de transmission verticale du VIH de 72.000 à 8.300 chez de nouveaux enfants infectés en Afrique du Sud tout au long 2015. Cependant, les obstacles culturels, des infrastructures, et socioéconomiques ont empêché la mise en route du protocole de prévention de la transmission mère-enfant (PTME). D’autre part, la recherche de solutions possibles pour éviter ces obstacles dans des milieux ruraux est spécialement limitée. Cette étude est orientée à l’identification des difficultés et des solutions pour la mise en route, adaptation et soutenabilité du protocole PTME dans des régions rurales d’Afrique du Sud. Dans ce but, l’étude a compris 45 interviews qualitatives, 12 group de discussion (n = 75) et un workshop de deux journées (n = 32 participants) développés avec la participation des directeurs de district, des cliniciens responsables, personnel d’aide et des patients de 12 cliniques rurales. La livraison et l’acceptation du protocole PTME ont été évaluées en accord avec le CFIR (Consolidated Framework for Implementation Research) : 15 sujets associés aux difficultés et aux solutions sont apparus. La révélation de l’entrainement dans le protocole PTME et du status sérique VIH sont des sujets caractéristiques de l’intervention. La disponibilité d’espace, le traitement et contrôle des histoires cliniques et la limitation de moyens humains peuvent être considérés comme des aspects moins directs, tandis que l’usage et la disponibilité de ressources matérielles, les relations entre le personnel qualifié et le patient, le transport et son horaire sont des aspects de contrôle plus directs. Les aspects qui sont d’avantage en rapport avec les caractéristiques individuelles de chaque patient incluent les relations avec le personnel qualifié, la visite initiale de soin prénatale, l’adhésion au protocole, la culture et les stigmas. L’éducation du patient, la livraison des résultats des tests et le compromis masculin sont les aspects le plus importants pour la mise en marche du processus. Quelques lacunes dans l’application des soins ont été identifiées dans des régions rurales. Néanmoins, l’information obtenue des participants qui ont utilisé the schéma CFIR a fourni des données très précieuses pour trouver des solutions aux problèmes de la mise en route du protocole PTME. Dans le but d’améliorer au maximum la prévention du VIH, il est très important de suivre de très près le protocole PTME pour appliquer les corrections, adaptation et soutenabilité nécessaires

Without treatment, global rates of mother-to-child transmission (MTCT) of HIV ranged from 20% to 45% (De Cock et al., 2000), though these rates were substantially reduced by Prevention of Mother-to-Child Transmission (PMTCT) strategies (De Cock et al., 2000; Johri & Ako-Arrey, 2011; Luo et al., 2007). In 2009, approximately 72,200 South African children were infected with HIV through MTCT (Joint United Nations Programme on HIV and AIDS [UNAIDS], 2011). Given the number of vertically infected children in South Africa (SA), UNAIDS implemented a plan to reduce new HIV infections in children to 8300 by 2015 (UNAIDS, 2011), reduce the rate of MTCT of HIV to less than 5%, and increase antiretroviral therapy (ART) uptake among infant-mother pairs to 90% (UNAIDS, 2013). In SA, national PMTCT programing resulted in a substantial reduction in MTCT rates in facility-based studies, though the impact of health system programing on these reductions is unclear (Goga et al., 2014).

PMTCT strategies are cost-effective in regions where HIV rates are high (Johri & Ako-Arrey, 2011). Since 2009, SA has achieved an increase in PMTCT accessibility (Mayosi et al., 2012), 90% PMTCT coverage (Peltzer et al., 2011), and a 52% decline in new HIV infections (UNAIDS, 2013). However, rural SA regions continue to report higher MTCT rates (Wettstein et al., 2012) as well as cultural, infrastructural, and socio-economic barriers that influence PMTCT availability and accessibility (Amnesty International, 2014; Ladur, Colvin, Stinson, & Thorne, 2015; Peltzer, Mosala, Shisana, Nqueko, & Mngqundaniso, 2007; Skinner, Mfecane, Gumede, Henda, & Davids, 2005). One such rural area is Mpumalanga Province, which has one of the highest antenatal clinic HIV prevalence rates (35.6%; National Department of Health, 2012) and second highest population-based prevalence of HIV in SA (14.1%; Shisana et al., 2014).

Barriers to PMTCT implementation in Mpumalanga include illiteracy, mothers’ unwillingness to test themselves or their infants, lack of government documentation, poor medical compliance (Peltzer et al., 2009, 2011), and patient dissatisfaction (Ladur et al., 2015; Phaswana-Mafuya et al., 2011). Patient-level barriers include poor nevirapine (NVP) uptake and nondisclosure of HIV serostatus despite intervention (Phaswana-Mafuya et al., 2012). Health system barriers include insufficient staff training, staff shortage, limited supervision (Ladur et al., 2015; Peltzer et al., 2009; Phaswana-Mafuya et al., 2012), inadequate patient tracking (Peltzer et al., 2009) and loss to care before completing the PMTCT protocol, the PMTCT cascade (Barker, Mphatswe, & Rollins, 2011). Drop-out from the PMTCT cascade has been associated with lack of on-site HIV testing, delayed HIV testing results, lack of HIV serostatus awareness, nondisclosure to partners and delayed ART initiation (Woldesenbet et al., 2015). These barriers to PMTCT highlight the need to identify challenges and devise solutions to enhance PMTCT protocol implementation in rural South Africa. Proposed solutions to these challenges have included peer support for PMTCT retention/adherence (Sam-Agudu et al., 2015), male partner involvement (Jones et al., 2014; Jones, Chakhtoura, & Cook, 2013; Peltzer et al., 2009, 2011), HIV testing (Peltzer, Mlambo, & Phaweni, 2010; Sprague, Chersich, & Black, 2011), ART initiation during pregnancy (Tsague et al., 2010), improved clinic staffing, infant follow-up post-delivery, intimate partner violence (IPV) screening, and promotion of safer infant feeding practices (Peltzer et al., 2011). Improved protocol implementation may also reduce MTCT among women who seek care late in pregnancy (Lallemant et al., 2015). However, uptake of the proposed solutions has been limited, and challenges continue to impact comprehensive implementation in rural communities.

Solutions geared to the local socio-cultural context may be more effective in responding to local barriers (Gourlay, Birdthistle, Mburu, Iorpenda, & Wringe, 2013) but have received little attention in PMTCT research. This study used a comprehensive implementation science approach, the Consolidated Framework for Implementation Research (CFIR), to evaluate the delivery of the PMTCT protocol and identify implementation barriers and novel, culturally tailored solutions. The CFIR is designed to maximize health outcomes by facilitating effective intervention implementation, and is comprised of five domains: Intervention characteristics, outer setting (e.g. patient needs and resources), inner setting (e.g. culture), characteristics of the individuals involved (e.g. agency, influence), and implementation process (Damschroder et al., 2009).

Utilizing the five CFIR domains and constructs, the influence of healthcare system-, clinic staff-, and patient-level challenges and solutions were examined at each healthcare system level on implementation and execution of the PMTCT protocol. It was theorized that in-depth examination of PMTCT protocol provision in rural SA could guide implementation, uptake, and sustainment of the program, thereby improving its effectiveness. It was reasoned that key stakeholders would provide the most valuable information on challenges, solutions, and strategies to enhance uptake of the PMTCT protocol in rural Mpumalanga Province, and that study findings could guide implementation in comparable rural regions.

## Method

### Participants and procedures

Prior to study onset, approval was obtained from the Human Sciences Research Council Research Ethics Committee, the University of Miami Institutional Review Board, and the Mpumalanga Department of Health and Welfare (provincial, district, sub-district, and clinic levels). This study was conducted in collaboration with Protect Your Family (PYF), a PMTCT initiative being administered at Community Health Centers (CHCs) in Mpumalanga Province. Information regarding PYF CHC selection has been published (Jones et al., 2014) and is detailed on clinicaltrials.gov (protocol NCT02085356).

#### Recruitment, enrolment, and compensation

Clinic staff interviews (*n* = 48) and patient focus group discussions (*n* = 12 FGDs) were conducted at CHCs, and small group discussions (*n* = 10; total *n* = 32 participants) were conducted at the HSRC in Pretoria, SA, among attendees at a workshop on dissemination and implementation of evidence-based interventions. Three to nine female patients attending the CHCs for ante- and post-natal care services were recruited for each of the FGDs. The consenting process took place in private CHC offices in English, Zulu, or Sotho. Interviews ranged from 1 hour to 1 hour and 24 minutes (mean = 1 hour and 9 minutes), and FGDs ranged from 23 to 55 minutes (mean = 41 minutes). FGD participants were compensated ∼US$5 in South African Rand for their participation; interviewed staff and workshop attendees did not receive monetary compensation.

HSRC study personnel, social scientists with doctoral, masters or bachelor degrees with specialization in HIV research, conducted all interviews, FGDs and facilitated the workshop. Rapport was developed with participants by engaging in casual conversation prior to the interviews and FGDs. Interviews and FGDs were audio-recorded, transcribed verbatim, and translated. FGD and interview transcripts resulted in 67,358 and 204,607 words, respectively.

#### Workshop

Workshop attendees were divided into small groups of clinicians and senior local stakeholders to facilitate the inclusion of different perspectives. Three stems were provided: (1) brain-storming challenges among attendees working in similar positions, (2) exploring solutions among a group of varied attendees, and (3) discussing clinic strengths. A group leader recorded challenges and solutions once consensus was reached and then reported their ideas to all other attendees. Themes identified during the workshop guided University of Miami and Human Sciences Research Council staff in developing topics for the qualitative interviews and FGDs. The workshop was conducted on 8 and 9 September, 2014.

#### Clinic staff interviews

Questions and stems for clinic staff interviews are presented in Table 1, and targeted information about the strengths, potential solutions, and challenges of HIV care at CHCs. The individual interview format was used to maximize staff disclosure regarding the topics presented, as it was believed that a group format would hinder staff disclosure when discussing certain topics with coworkers or supervisors. Interviews were conducted from 5 November 2014 to 27 May 2015.

**Table 1.** Interview questions and stems for qualitative interviews.

**Training, protocol, and clinic environment**
1. Describe the training you received to care for people living with HIV. What were the most useful aspects of your training? What kind of ongoing training have you received? When was the last training? Do you wish you received more training on HIV care and PMTCT? Why? How do you feel about the training you have received? What skills do you use to care for people testing HIV positive during pregnancy?
2. Describe the PMTCT protocol at your clinic. What are staff attitudes about providing the protocol? What challenges are there in providing the PMTCT protocol? What gaps are there for care?
3. Describe the environment at your clinic.How do staff work together?
4. For patients who test positive, describe how they receive their results? What efforts are made to get male partners tested for HIV also? For patients who test positive for HIV, how are they engaged in treatment? What efforts are made to get them engaged in treatment?
5. At what stage in pregnancy would you like women to begin attending the ANC? When do women typically begin attending? Why at that time?
6. For women attending the ANC, what efforts are made to get partners to attend? What are staff attitudes about men attending the ANC? Where do they wait? Do they come in the room during the woman’s visit?
7. Describe some of the challenges experienced by staff in implementing the PMTCT protocol? What do you find challenging about getting women and their partners to test for HIV? What do you find challenging about the PMTCT program? What elements of the protocol are the most challenging?
8. Can you describe a time when all or part(s) of the protocol was not followed or not working at your clinic or at another? Why was the protocol not followed? What happened?
9. What changes would you recommend to ensure that the protocol for PMTCT is implemented?
10. What are some of the barriers that prevent making these changes to improve the program? What would need to be in place for these changes to happen and work well? Describe what you think could be barriers to adopting these changes at your clinic or at any clinic. How ready are staff to change if it would improve the PMTCT program? What would need to happen to help staff get ready for change?
11. Is there anything you would like to add or think would be useful to know in improving the implementation of the PMTCT protocol and achieving its goals?
**PMTCT goals and potential solutions** The following are some problems that occur in clinics. What kind of solutions are used? GOAL: Step 1: Early ANC booking (<20 weeks), counseling, HIV testing and CD4 testing
Many mothers come late for their first antenatal booking Not all mothers are counseled and tested for HIV testing at their first antenatal booking Not all HIV-positive mothers have CD4 test blood drawn GOAL: Step 2: Treatment for patients w/CD4 > 350
Mothers have CD4 test drawn, but do not return for results Mothers are often delayed before their receive ARV/HIV medication waiting for CD4 count results Step 3: CD4 < 350: rapid referral and HAART initiation
HAART clinics are overburdened and pregnant women are delayed in starting ARV/HIV medication Clients are referred for ARV/HIV medication but do not pitch up at ARV clinic Clients are referred for ARV/HIV medication but no information is sent to the patient, which leads to delays and duplication Clients are delayed for ARV/HIV medication because a treatment supporter has not been identified GOAL: Step 4: Labor ward: three-hourly AZT during labor, sdNVP to mother and baby, and start AZT to infant
The delivery of PMTCT medicines is unreliable during labor It is not always clear which mothers are part of the PMTCT Program Some mothers did not get tested during the ANC period but can still receive ARV for PMTCT GOAL: Step 5: HIV exposed babies get PCR at 6 weeks
The post-natal care clinic does not always know which babies were HIV exposed. PCR testing is not always reliable.
GOAL: Adherence, exclusive breastfeeding, male involvement, family planning, reporting & data capture Some mothers may feel babies do not get enough nourishment only breastfeeding Sometimes individual patient information is not correctly or not at all reported in registers and reporting templates – monthly summaries. Some women may not take their ARV medications as prescribed. It is difficult to involve the male partner in PMTCT

#### Patient focus group discussions

FGD questions and stems addressed testing, HIV prevention, treatment, infant feeding, family planning, safer sex, male involvement, personal experiences with PMTCT and HIV knowledge (see Table 2). FGDs were conducted with mothers and expectant mothers with HIV. A group format was used with patients to allow discussion among patients, which, given the variety of issues faced by patients, theoretically provided a broader perspective of challenges and solutions. FGDs were conducted from 18 February 2015 to 7 May 2015.

**Table 2.** Interview questions and stems for focus groups.

**Acceptability**
1. What have you heard about antenatal care and the PMTCT program at the antenatal clinic (ANC)? What seems to work well in the ANC PMTCT program? What does not work well in the ANC PMTCT program? What could be changed to improve the PMTCT program? How could the ‘flow’ of services be changed to improve the program? What issues in the community affect the way PMTCT programs are provided? How could the community strengthen or improve the program? What are some other issues that affect the way PMTCT programs are provided? What have you heard about the Vikela Umndeni project? Describe what you know about the project.
**Fidelity**
2. What have you learned about the components of the PMTCT protocol from the clinic staff? These components include testing, HIV prevention, ARV treatment, infant feeding, family planning, safer sex and involving your partner in your pregnancy. What have you heard about the amount of time patients spend at the clinic during pregnancy? When do women come for antenatal care/pregnancy care for the first time? What kind of experiences have people had with obtaining their test results promptly? What experiences do people have in receiving their ARV treatment? How can services be improved? What else could be done in the way of new programs, like Vikela Umndeni?
3. What have you heard about communication between patients and the health care staff at the clinic? What have you heard about communication between patients and the health care staff at the clinic? How does communication affect receiving health care during pregnancy? What kinds of changes could improve communication?
4. What have you heard about staff appearing fatigued (worn out, tired) or burned out (less interested in work) with patients? How does staff fatigue or burn out affect the way provide health care? How does staff fatigue or burn out affect new programs, like Vikela Umndeni? What kinds of changes could reduce the staff burden?
**Coverage**
5. What have you heard about staff appearing fatigued (worn out, tired) or burned out (less interested in work) with patients? Are some clinics more popular than others? What makes them better or worse? Why did you choose this clinic for your care during pregnancy? If you attended a different clinic, why did you choose that other clinic?
6. The following are some problems that occur in clinics. How could these problems be solved? Many mothers come late for their first antenatal booking. Not all mothers are counseled and tested for HIV testing at their first antenatal booking. Not all HIV-positive mothers have CD4 test blood drawn. Mothers have CD4 test drawn, but do not return for results Mothers are often delayed before their receive ARV/HIV medication waiting for CD4 count results. HAART clinics are overburdened and pregnant women are delayed in starting ARV/HIV medication. Clients are referred for ARV/HIV medication but do not pitch up at ARV clinic. Clients are referred for ARV/HIV medication but no information is sent to the patient, which leads to delays and duplication. Clients are delayed for ARV/HIV medication because a treatment supporter has not been identified. The delivery of PMTCT medicines is unreliable (not always done) during labor The post-natal care clinic does not always know which babies were HIV exposed Some mothers may feel babies do not get enough nourishment only breastfeeding and may mixed feed their babies. Some women may not take their ARV medications as prescribed. It is difficult to involve the partners in PMTCT, in some cases, men are not involved.

### Qualitative analyses

Grounded Theory (Glaser & Strauss, 2009) was used for coding and analyzing interview, FGD, and workshop data (Damschroder et al., 2009), which were coded and analyzed closely to identify common themes related to barriers and solutions to PMTCT implementation. The coding strategies used to code all transcripts included open, axial, selective, and theoretical coding (Glaser, 2005; Strauss & Corbin, 1990). During the coding process, an external coder was trained and asked to code five previously coded transcripts to assess the level of agreement and reliability of identified themes and interpretations. The same procedure was repeated with a third coder and fourth coder, with fewer transcripts (three). Coding and thematic disagreements, although uncommon, were discussed until consensus was reached. In addition, regular meetings were conducted with the team to discuss and redefine codes and themes, which were followed by reflections on the influence of perceptions and assumptions on coding. Interview, FGD, and workshop themes were compared and contrasted using theoretical memoing (Glaser, 1998) to identify different staff- and patient-level perspectives for interpretation and reflection by the coders and authors.

## Results

Of those approached for interviews (*n* = 60, 5 per facility) and FGDs (*n* = 120, 10 per CHC), 80% and 63% agreed to participate, respectively. The reason provided by those declining was primarily time constraints. Interview participants included lay counselors, facility operation managers, clinic facility managers, professional nurses, assistance nurses, staff/enrolled nurses, and an HIV Counseling and Testing counselor. The workshop was held with 32 clinicians and stakeholders, including facility operation managers, clinic committee members, and district and sub-district HIV, AIDS, and sexually transmitted infections (HAS) managers; all participated. Fifteen themes emerged from interview, FGD and workshop data (see Table 3). Emerging themes are presented under the five CFIR domains to identify the challenges and solutions associated with each of the factors that have been found to be predictive of implementation success or failure (CFIR, 2014; Damschroder et al., 2009). It was theorized that organizing each of the themes by CFIR domains would help future programs, interventions, and policies target and prioritize areas of need, while validating the domains as predictors of implementation success or failure.

**Table 3.** Themes categorized by Consolidated Framework for Implementation Research (CFIR) domains.

Theme	Summary	
	Challenges	Solutions
**Intervention characteristics**		
1. PMTCT training	Continuous additions and changes to PMTCT protocol that staff are not always informed about.	Need for a continuous training to match the frequency of changes in protocol.
2. Disclosure to family and partners	Fear of losing support upon disclosure of HIV serostatus. Mixed feeding results from nondisclosure to infant caretakers. Low rates of condom use, potentially increasing the risk of re-infection.	Decreasing fear regarding potential negative reactions to disclosure with current support system. Promote the use of peer education and mentorship to facilitate stronger bonds with support system. Promote disclosure of HIV serostatus as men are more likely to use a condom when they are of aware of their partner’s serostatus.
**Outer setting**		
3. Facility space	Limited facility space to meet patient demands affecting patient privacy and attendance, and male involvement. Increased risk of airborne infections in crowded spaces.	Increase facility space. In the absence of financial resources, maintaining appointment logs to limit to the number of patients seen simultaneously, or providing HBC.
4. Patient health record management	Poor patient tracking due to human error, lack of resources, and participant misreporting.	Improve patient tracking through HBC workers, or implementation of electronic medical record.
5. Clinic staff shortage	Staff overburden and patients leaving the clinic without being seen or treated.	Increase patient outreach, create mobile clinics, modify staff schedule to have more personnel on busier days, increase staff productivity, and improve patient scheduling.
**Inner setting**		
6. HIV testing and medication supply use and availability	Shortages and lack of supplies to complete PMTCT procedures resulting in late detection of pregnancy and HIV, late onset of treatment, and unprotected sex.	Continued reliability on other clinics for needed supplies, or working with pharmaceutical distributors for planning of needed supplies.
7. Staff–patient communication and relationship	Factors affecting patient attendance to clinic for services and barriers to staff–patient communication (e.g. staff attitude and temperament).	The potential role of improving staff attitude as a way to increase patient uptake of clinic services.
8. Transportation and scheduling	Personal safety concerns, lack of transportation, and poor availability of emergency services during labor.	Increasing the availability of services and resources available to women during pregnancy and labor to serve the transportation needs of clinic patients.
**Characteristics of individuals**		
9. Professional relationships among staff	Factors affecting and impeding a collaborative working environment.	Increasing mentorship and supervision. Conduct evaluations of staff performance which include recognition.
10. Initial ANC visit	Misunderstanding of pregnancy among patients, lack of motivation, and inadequate understanding of PMTCT guidelines.	Providing information on how to identify pregnancy earlier, and increasing motivation by emphasizing potential benefits.
11. Adherence to PMTCT treatment	Poor social support, medication side effects, lack of education and understanding of the PMTCT protocol, cursory or inadequate explanations of treatment instructions.	Increase attendance to and awareness of support groups; building a therapeutic alliance between the patient and provider.
12. Culture and stigma	Cultural, community misconceptions, and societal beliefs affect PMTCT implementation and uptake.	Increasing level of comfort for patients at the clinic, as well as raising community awareness and education to dispel HIV myths and misconceptions.
**Implementation process**		
13. Patient counseling and education	Patient dissatisfaction with clinic services, and unfamiliarity with support group services.	A need for promotion of support group availability by clinic staff.
14. Delayed reporting of CD4 test results	Delayed CD4 count testing as a result of laboratory delays in releasing results, lack of supplies, and misplacement or erroneous delivery of results.	Increasing reliability of messenger services and patient outreach, and when feasible, provision of on-site CD4 testing and results.
15. Male involvement	Male involvement is affected by many factors, such as traditional perceptions of pregnancy, clinic schedules conflict with male partners’ work schedules, and limited clinic space to accommodate male partner attendance.	Better outreach and education aimed at male partner engagement, such as involving more men PMTCT service provision, and dispelling the notion that pregnancy is only a woman’s issue through male peer interaction.

### Intervention characteristics

The CFIR domain of intervention characteristics addresses whether an intervention – the PMTCT protocol – is internally or externally developed, as well as the perception of the intervention by key stakeholders, intervention credibility and the potential for positive health outcomes. Within the CFIR, intervention characteristics, e.g. intervention source, intervention quality, relative advantage of intervention, adaptability, complexity, indicate the potential for PMTCT to demonstrate effectiveness, considering stakeholder perceptions and the pros and cons of implementation (CFIR, 2014). PMTCT training, disclosure of HIV status to partner/family and condom use emerged as the most influential themes in intervention characteristics.

#### PMTCT training

*Challenges.* Among clinic staff, the majority expressed satisfaction with the length and content of PMTCT training received, felt confident about the skills they had gained, and understood PMTCT. Participants reported gaining interpersonal skills and learning the PMTCT protocol, which helped them during patient interactions:

The skills that is more important are interpersonal skills, in terms of counseling and then calming the patient down so that they can understand and accept that HIV will be part of their life … (Professional Nurse, Clinic J)

Some clinic staff appeared confused regarding PMTCT, perhaps due to the amount of time elapsed since initial training. This confusion underscored the benefit of regular follow-up/refresher training sessions:

I don’t remember [when asked about PMTCT] but I do have trainings that involve HIV. (Professional Nurse, Clinic R)

Some staff shared that nurses at various facilities may have different levels of understanding regarding the protocol, which may lead to incorrect application of PMTCT procedures:

… there are times where you feel there new drugs that are introduced or there are new blood that supposed to be done but you don’t know you just sticking to the old guidelines. (Professional Nurse, Clinic Y)

*Solutions.* Most participants (79%) desired additional training to remain current on PMTCT guidelines. Workshop attendees and clinic staff agreed that providing monthly or quarterly refresher trainings and mentoring/coaching following major updates to the PMTCT protocol guidelines would enhance implementation. One clinic staff member suggested that PMTCT protocol changes should be provided to all clinics immediately:

There must be refresher trainings at least twice a year. (Lay Counselor, Clinic S)

We must always be updated if there is something new. (Professional Nurse, Clinic P)

We should have mentors … . (Professional Nurse, Clinic H)

An assistant nurse suggested that Home-Based Care (HBC) personnel should be trained in PMTCT; HBC workers monitor and control the spread of tuberculosis (TB) by visiting patients at home, a model that could be implemented with PMTCT patients:

Women to do home visits so that they can check if the patients are taking their pills or not because other don’t take their pills and it becomes a challenge. (Assistant Nurses, Clinic O)

#### Disclosure to family and partners

*Challenges.* Failure to disclose HIV status was a major barrier to receiving PMTCT care; in many cases, mothers left their babies with caretakers unfamiliar with PMTCT protocol:

Sometimes you leave your child at home and no one knows that you’re HIV positive and they start mix-feeding the child because you didn’t disclose your status. (FGD, Clinic O)

Most FGD participants reported difficulties disclosing their HIV serostatus. Many feared others would treat or perceive them differently, and 25% of participants feared abandonment or IPV by their partner:

Sometimes it is difficult [to disclose] because you don’t know how the person would react, because maybe the person would kill you or leave you … I was relying on him to buy me food. (FGD, Clinic J)

Many FGD participants acknowledged not consistently using condoms (58%) due to nondisclosure, misinformation, and inability to influence male partners, who were commonly identified as decision-makers for condom use. FGD participants repeatedly asserted that condomless sex increased the possibility of infection/re-infection, which encouraged many couples to use condoms:

… they [nurses] have also taught us about HIV and how one can get infected, and also told us that if we are both HIV positive we shouldn’t stop using condom because we are going to re-infect each other … and the doctors will no longer be able to control it. (FGD, Clinic O)

*Solutions.* FGD participants and staff asserted that addressing women’s anticipation of negative post-disclosure reactions may promote an environment conducive to disclosure. Workshop attendees suggested that women should identify mentors to guide them pre- and post-natally, as well as through the process of disclosure. Addressing clinic- and individual-level challenges to male involvement prenatally could increase societal acceptance of men in the antenatal process and help women build stronger bonds with their partners, which may decrease fears surrounding disclosure and increase male partner support:

When I arrive at home from the clinic, he asks how it went at the clinic and when I arrive with treatment I explain to him what it is for and how is going to help the baby and me. Even after birth it is going to be easy to give the baby treatment because he knows the situation. And even when you explain how long the baby is going to be on treatment, he is going to understand. And he must also know about breastfeeding and he must know about how the baby takes the treatment. (FGD, Clinic P)

It was also suggested that promoting disclosure of HIV serostatus could effectively combat inadequate condom use among HIV affected couples:

It’s important [to disclose] because if he doesn’t know he’ll refuse to use a condom but if he knows our statuses he’d understand he should follow the rules. (FGD, Clinic K)

### Outer setting

The CFIR outer settings domain addresses patient outreach, networking, accessing/providing resources, cosmopolitanism, peer pressure, external policy, and incentives (CFIR, 2014). Facility space, patient health record management, and clinic staff shortage emerged as outer-setting themes.

#### Facility space

*Challenges.* Clinic staff (32%) felt that their facility was too small to meet patient demands, which contributed to problems with patient privacy – e.g. multiple patients being seen simultaneously – and with individual and group counseling. The clinics’ physical infrastructure also presented a major challenge to male partner involvement in PMTCT.

If maybe there is two ladies there [labor room], we don’t allow them [male partners] but they are scared of clinic, let alone to hear what we are saying. (Facility Operation Manager, Clinic K)

… one-to-one communication is deprived. We have to screen around in between patients and some don’t feel confident to talk. (Professional Nurse, Clinic N)

Patients sometimes waited outside due to limited space and left the queue without being seen, resulting in some patients not returning for care. In some cases, patients attended a different clinic and, due to the lack of a transferrable record, medical histories were unavailable. Generally, in all clinics and CHCs, one day is set aside for ANC visit, which limited operating time and led to clinic congestion. However, ANC clinic days were an effective strategy for minimizing the risk of contracting airborne diseases, such as TB, which arose from mothers standing in a mixed-reason-for-visit clinic queue:

I don’t think it’s a right thing for babies to be mixed with TB patients; it doesn’t make sense to me because the children inhales TB quickly. (Lay Counselor, Clinic P)

*Solutions*. Some clinics circumvented problems associated with the formation of mixed-reason-for-visit clinic queue by immediately treating patients who outwardly exhibited signs of contagious infections. It was also suggested that infants could be treated on specific days to minimize exposure to airborne diseases:

If we notice that a person is coughing and has TB without being screened, we make sure that we remove that person from the queue … (Lay Counselor, Clinic P)

Babies don’t have their own day at the clinic so it becomes a problem. (Lay Counselor, Clinic P)

In addition to increasing facility space, it was suggested that clinics could benefit from maintaining appointment logs instead of having an open, set schedule (i.e. one six-hour ANC day) providing PMTCT counseling and education using HBC.

#### Patient health record management

*Challenges*. Forty-four percent of clinic staff and workshop attendees reported multiple errors related to health record management, influenced by both patients and staff:

… they [staff] don’t mark on the child booklet whether the mother was HIV positive or not. (Professional Nurse, Clinic N)

We do have human errors … maybe there is a line and I have to mark patient 10, but I make a mistake and mark patient no.11. (Professional Nurse, Clinic J)

Files are getting misplaced or lost because we have only one data capturer … (Professional Nurse, Clinic W)

… those young mothers, first they don’t take care of the baby’s clinic card, sometimes they lose the cards … (Assistant Nurse, Clinic J)

*Solutions.* Many clinics had procedures in place to prevent and abate errors related to health record management, such as training and meetings. If the patient destroyed their summary card to hide their serostatus, staff had the information recorded in their records:

We also write in the post antenatal history so this book will tell me even if the patient destroyed the card. (Professional Nurse, Clinic K)

Double-checking data entries was recommended to prevent errors, and an assistant nurse suggested use of a national electronic medical records (EMR) to help improve patient tracking.

#### Clinic staff shortage

*Challenges.* Half of participants (56%) reported that long wait periods due to staff shortages and increasing patient census discouraged ANC attendance. Clinic staff from multiple clinics reported that they might see up to 6500 patients in a given month with only 3 sisters available on a given day:

There were only 3 Sisters, and patients end up going home around 6pm. (Professional Nurse, Clinic J)

Staff shortages prevented some staff from performing home visits. Because women who were later in gestation were attended to by staff before women earlier in gestation, staff shortages also contributed to women’s untimely access of ANC.

That’s why they don’t come early … the patients will complain and think it is better to come late for booking … because when they come early they don’t get help (Lay Counselor, Clinic N)

*Solutions.* Workshop participants suggested that in addition to more personnel, clinics would benefit from having more staff on busier days and from scheduling patient-specific appointments. Clinic staff and workshop participants suggested hiring more staff and creating mobile clinics to reduce staff shortages, theorizing that waiting times would decrease and increase patient satisfaction:

Add more clinics and add more staff to those facilities and have mobile clinics for outreach … (Professional Nurse, Clinic X)

### Inner setting

The CFIR inner-setting domain includes all aspects of the patient experience (CFIR, 2014): clinic service hours, clinic staff communication and interaction with patients, patient testing, patient education and support services, and clinic-directed healthcare campaigns. The themes, e.g. HIV testing and medication supply availability, quality of communication or interaction between staff and patients and availability of clinic transportation for patients, were related to the inner-setting domain.

#### HIV testing and medication supply use and availability

*Challenges**.*** Thirty percent of staff reported test kit, condom, medication shortages. Clinics order their medical supplies from pharmaceutical distributors and shortages experienced by the distributors affected the entire province.

We sometimes have a shortage of testing kits both for pregnancy and for HIV. (Facility Operation Manager, Clinic K)

… last year we stayed plus minus 3 months without condoms. (Lay Counselor, Clinic X)

For newborns, sometimes there’s no NVP. Then we have use lamivudine and others don’t understand how to replace the treatment. (Professional Nurse, Clinic J)

*Solutions.* Many clinics dealt with supply shortages by borrowing supplies from neighboring clinics and hospitals, although this was not a permanent solution during periods of regional shortages:

If we don’t have those specimen things we ask our next clinic like neighbors … (Professional Nurse, Clinic N)

Workshop participants asserted that supply shortages could be prevented by working with pharmaceutical distributors for stock planning at the district- and province-level. One clinic nurse confirmed that her clinic did not experience the same shortages as other clinics because it planned its stock beforehand, using the clinic patient load as a reference:

Planning can assist by making sure that we have enough equipment and resources to do the job, like for an example, having all the necessary material like stationery and stock medication. (Professional Nurse, Clinic Y)

#### Staff–patient communication and relationship

*Challenges.* Most FGD participants asserted they communicated well with clinic staff. Three participants reported that fair treatment and clinic reputation influenced their decision to attend a clinic.

I chose this clinic because I think it is right and I always hear my friend and my siblings’ say that the sisters treat people good, so that is why I chose to come here. (FGD, Clinic S)

Three FGD participants stated that some staff were more approachable than others, which may lead the patient to leave the clinic without being seen. Participants in one FGD spoke of the need for patience in interacting with clinic staff. Being short tempered with clinic staff could lead to conflict, which could result in the patient not receiving treatment or bringing their infants post-natally:

If you are short tempered you won’t get help here and you will end up exchanging words with the nurses or even in a fight with the sisters. You end not coming to the clinic anymore and even for fetching your medication and also end up not bringing the baby to the monthly visits. (FGD, Clinic J)

*Solutions.* Clinic staff and workshop participants asserted that improving patient and staff satisfaction would greatly benefit PMTCT implementation and uptake, since more patients would follow staff recommendations and PMTCT guidelines if staff had a positive attitude:

We have positive staff … all of us we work well together to win a patient so that at end the patients would do everything that we want the patient to do. (Assistant Nurse, Clinic O)

#### Transportation and scheduling

*Challenges.* One-third (33%) of FGD participants had trouble with transportation and obtaining antenatal services. In some cases, women lived far from the clinics, which hindered support group attendance. Lack of transportation led to home births*.* Ambulance drivers may delay arrival if the woman is not a first-time mother:

When you call [the ambulance], they would ask you if it’s the first you have had a baby or not, and if you say it’s not the first time they’ll take their time to come because they think you already know what to do, that’s how it is. (FGD, Clinic H)

*Solutions.* Given that some participants reported that ambulance drivers provided more timely services to first-time mothers, it was suggested that increasing the availability of services and resources, as well as changing attitudes towards pregnancy and labor among healthcare workers, could address challenges associated with transportation.

I think it would be better us to have standby ambulances always when we need them. (FGD, Clinic H)

### Characteristics of individuals

The CFIR domain of characteristics of individuals refers to organizational relationships and communication. Four themes in this domain were identified: professional relationships among staff, initial ANC visit, PMTCT treatment adherence, and culture and stigma.

#### Professional relationships among staff

*Challenges*. Two-thirds of clinic staff (65%) felt that professional relationships at their clinic were positive. Others felt their relationship with team members was ‘cooperative,’ involving frequent discussions about work-related matters. Positive professional communication facilitated mentoring among staff who had not been formally trained and had to rely on coworkers for information:

Our relationship is good, we work as a team especially when it comes to PMTCT, so I haven’t seen anything bad because we always ask if there is a problem. (Nurse, Clinic X)

Collaboration and teamwork between staff members at clinics was seen as positive and could be mobilized and extended to community partners to improve patient outreach:

The stakeholders, traditional healers and religion … it would be better if we are working along with them because they would understand better. (Nurse, Clinic S)

Staff conflict related to lack of communication, training misinformation, staff shortages, and perceived inequities based on job designation was reported in 35% of the clinic staff interviews. One lay counselor shared that her position was looked down upon by management:

… we used to have lay counselor meetings and they would tell us to move so that other people can use the space, and when we want to apply, we’re not recommended. I wish that the management could treat us as human beings. (Lay Counselor, Clinic S)

*Solutions.* Clinic staff suggested that if supervisors worked directly with patients, management would learn to appreciate the work of lower-level staff:

… they [supervisors] also need to see when there are patients what do we mean when we say there’s shortage of staff. (Professional Nurse, Clinic S)

Workshop participants noted that many employees lacked motivation and felt unappreciated, and suggested conducting objective performance evaluations and offering incentives via monthly recognitions, praise, and other forms of reinforcement.

#### Initial ANC visit

*Challenges*. Staff (78%) reported that patients typically started their first ANC visit after 20 weeks gestation, and 42% of FGD participants reported that they or someone they knew attended their initial ANC visit after 20 weeks. Reasons for late entry to ANC included the mother not realizing she is pregnant, lack of motivation, inadequate understanding of PMTCT guidelines, and unfriendly staff attitudes.

My tummy was right and I was normal and my body was normal, no changes, and I did not show that I was pregnant until delivery. (FGD, Clinic W)

When you come to fetch the treatment, they treat you better, unlike coming for the first time. (FGD, Clinic J)

*Solutions.* Based on the challenges described by participants, it was suggested interventions to increase motivation to participate in ANC and knowledge about identifying pregnancy, and additional staff training, would increase uptake and implementation of PMTCT. One FGD participant suggested specialized for pregnant WLWH to increase comfort with the initial ANC visit:

They mix us with those that are HIV negative because we enter here first so that we won’t feel sidelined. At first they used to take us to the other room so people were laughing at us and other patients stopped coming to the clinic because they were being laughed at. People used to gossip when they saw you entering the room and they go around telling people at the township … (FGD, Clinic Y)

#### Adherence to PMTCT treatment

*Challenges*. FGD participants reported dizziness, vomiting, nightmares, and weight gain as side effects contributing to ART nonadherence:

I was feeling sick and dizzy every time after drinking them. (FGD, Clinic 2)

You’d find that women don’t drink their pills and she would tell you that she vomits from drinking the pills and the pills cause her to be bodily confused. (Assistant Nurse, Clinic O)

Poor understanding of the PMTCT protocol were cited as reasons for nonadherence by clinic staff. Some women became nonadherent when staff did not explain certain procedures:

[With regard to mixed feeding] She was in a hurry, so she didn’t explain everything to me because she was busy. (FGD, Clinic W)

Many nurses reported that women were more likely to default if they did not have a support system. Nondisclosure and denial of HIV serostatus also led to decreased participation in support groups.

You must calculate the pills and it is just to see if she adheres to treatment. You will be surprised and ask her why she is left with 5 pills not 1 pill and she will answer by saying, ‘Sister I haven’t accepted my status and I didn’t take them up until I have accepted 5 days later and that is when I decided to take the treatment.’ (Professional Nurse, Clinic S)

*Solutions*. Staff felt that the most effective way to increase patient’s PMTCT protocol adherence was education, such as individual testing and treatment counseling. Staff also suggested meetings and health talks at churches and non-governmental organizations (NGOs) to promote adherence and knowledge at the community level to help eliminate misconceptions and stigma, and foster a more supportive atmosphere/culture for PMTCT patients:

We need to educate the community about the importance of coming early to the clinic and the importance of taking the treatment … The community and other people who educate like churches and some NGOs should go to the community halls and educate people about PMTCT. (Professional Nurse, Clinic S)

#### Culture and stigma

*Challenges.* Culture and stigma were major treatment barriers for patients with HIV. Participants reported that older generations utilized traditional medicines and struggled to accept what they perceived as modern methods. HIV myths and misconceptions were commonly reported:

Sometimes if you tell a person that you are HIV positive, they say there is no such thing, all you have to do is buy Stametta [a South African herbal drink] and it will clean your blood. (FGD, Clinic R)

… my mother was very upset and my father kept saying that they are lying at the clinic. [My father] said ‘my child, these people are lying’ … and he kept saying ‘these people are lying to you, look at you … you are fit and healthy, you are not sick.’ (FGD, Clinic W)

Some clinic procedures perpetuated negative societal views of HIV, such as having separate lines for WLWH, which discouraged patients from attending clinic:

They are afraid of what would people say when they see her queuing the HIV positive patients queue. (FGD, Clinic K).

Widespread misconceptions affected adherence to the PMTCT protocol. One FGD participant shared that exclusive breastfeeding might not be the best choice for her baby, and that other mothers also shared this belief:

Sometimes I hear other mothers say that their babies don’t get enough by only breastfeeding. (FGD, Clinic W)

*Solutions.* In response to patients’ discomfort and to stigma associated with picking up ARVs, one FGD participant suggested that all patients, regardless of HIV serostatus, should retrieve their medication in private rooms. Community outreach, health talks, pamphlets, and media campaigns were suggested to reduce HIV-related stigma:

I want them to do something where if you came to fetch your pills, you must get inside the room and get everything. (FGD, Clinic Y)

They [the community] should be taught that HIV doesn’t infect easily, because other people think that if you drink from the same cup you are going to be infected and even if you touch them they think that you will infect them … (FGD, Clinic R)

### Implementation process

The implementation process is the broadest CFIR domain and refers to the overall planning, execution, involvement, and evaluation of the protocol (CFIR, 2014). Three themes in the PMTCT implementation process were identified: Patient counseling and education, delayed reporting of CD4 test results, and male involvement.

#### Patient counseling and education

*Challenges.* Although several participants were satisfied with clinic services, some FGD participants believed that clinic staff needed more PMTCT training, that counseling should incorporate nutritional guidance, and that staff must inform patients of PMTCT related support groups:

… they [the nursing staff] have spirit of Ubuntu and they talk to us with patience. (FGD, Clinic K)

… we don’t feel we are needed and the way they talk to us is like they lack knowledge about HIV. (FGD, Clinic J)

They don’t tell us what to eat. They only thing they tell us is to exercise. (FGD, Clinic Y)

*Solutions.* Although most participants were happy with the services provided by clinic, some wished that more staff would be available and that staff receive more training:

I wish government could hire more staff so that the queues can be less, at least have more staff. And in terms of the staff attitude, I am very satisfied in this clinic. We do get the service and sometimes patients come in numbers and the nurses are few and there is nothing they can do. (FGD, Clinic O)

I think the nurses still need workshop. (FGD, Clinic J)

#### Delayed reporting of CD4 test results

*Challenges*. One-third of clinic staff reported delays in laboratory test results due to laboratory delays in releasing the results, results being sent to the wrong clinic, lack of testing supplies, and limited laboratory schedules. Because of laboratory scheduling, patients struggled to take time off work, or were required to return a second time to have a CD4 test blood draw. In rural communities, time, long distances, and transportation cost were cited as challenges. Patients might also be asked to return on another day if testing supplies are not available:

… pregnant women come late after hours and then at lab they said we can’t take CD4 count … the other challenge is that they don’t provide enough lab forms or bottles for CD4 count, blood bottles. (Professional Nurse, Clinic O)

The delivery people deliver our results to another clinic, and deliver the other clinic’s results to our clinic. The only problem that I have noticed happening is: when we get their results, we send them to their clinic but they don’t return ours. (Lay Counselor, Clinic X)

*Solutions.* Interviewees and workshop attendees reported that laboratory challenges could be mitigated by providing all services at the same clinic where patients are seen. Staff suggested that more comprehensive patient outreach may be needed to remind and motivate patients to attend their appointments.

#### Male involvement

*Challenges.* A major challenge to ANC and PMTCT care was lack of male partner involvement. Although many clinics encouraged men to accompany their partners to the clinic, most reported that men did not attend the appointments. One clinic reported that only ‘younger men and foreigners’ accompanied their partners to their appointments, while older and local men did not. FGD participants reported that male partners were tested elsewhere, e.g. workplace, as opposed to being tested at the same clinic as their partner, and then may hide their status from their partner:

My partner went to the clinic and got tested secretly without telling me and he found out that he was HIV positive and they gave him treatment. Then he hid it from me until one day I noticed that there is a change in his body. (FGD, Clinic J)

It is not easy to involve them because most of the times they are at work (FGD, Clinic C)

Many staff and FGD participants reported that their partners were unable to attend clinic appointments due to schedule conflicts, dislike of long waiting periods, fear or shyness, and lack of space:

Men don’t like to wait, they want fast service. (FGD, Clinic N)

They are shy. (FGD, Clinic W)

Even though our rooms are small we made sure that he spent 3 to 4 hours with his partner before delivery. (Professional Nurse, Clinic K)

One staff member suggested that the lack of partner involvement resulted from extramarital relationships, as men did not want to be seen with a pregnant partner. Others suggested that men’s role as the head of the household influenced their involvement in treatment:

If the woman says, ‘Let’s go to do the test,’ they deny, but if the woman tested negative, he assumes that he is also negative. And number two, if the woman tested positive and she is on treatment, then the man steals the treatment. Those are the challenges. (Professional Nurse, Clinic O)

*Solutions.* Facility staff frequently encouraged men’s support and involvement in ANC, suggesting that better outreach, education, and involving male nurses in treatment would increase male partner support. Involving others emotionally close to the patient, not just partners, was also suggested.

Some clinics addressed the lack of partner involvement by creating a system by which women were retested with their partners, despite already knowing the woman’s status, to enable the couple members to receive their results together and to be counseled by a staff member. In some cases, men would not attend the clinic or listen to their wife without being specifically asked by staff to come in. Community outreach and education could also be used to help dispel the notion that only women can play an active role in pregnancy, as well as eliminate fears that might inhibit men’s involvement.

The sister asked him to come to the clinic because he didn’t understand why I should start the treatment, so they have called him here and counseled him, then eventually understood. (FGD, Clinic P)

## Discussion

This study addressed barriers and solutions to enhance implementation, uptake, and sustainment of the PMTCT protocol in rural SA. Using the CFIR domains (CFIR, 2014; Damschroder et al., 2009), themes related to the intervention characteristics domain included PMTCT training, and disclosure to partner and family. Facility space, patient health record management, and clinic staff shortage were themes of the outer-setting domain, whereas HIV testing and medication supply use and availability, staff–patient relationship communication and relationship and transportation and scheduling were themes of the inner-setting domain. Themes related to the domain of individual characteristics included professional relationships among staff, initial ANC visit by patients, adherence to PMTCT treatment and culture and stigma. Finally, patient counseling and education, delayed reporting of testing results, and male involvement themes comprised the implementation process domain.

Challenges identified in this study were consistent with those previously reported, e.g. high rates of patients lost to follow-up (Barker et al., 2011), poor adherence, staff shortages, and insufficient supervision (Peltzer et al., 2009). Confusion and uncertainty about frequently changing PMTCT protocols was common. Hierarchical relationships between staff prevented many staff members from learning about the PMTCT protocol firsthand and contributed to tension between staff and patients. Refresher training and mentoring were suggested would be useful to establish on-site experts as contacts for questions concerning the protocol. Staff could be retrained and evaluated regularly to ensure awareness of protocol changes and are providing the best care possible. Similarly, continuous quality improvement strategies are needed to identify and address new challenges and implement novel solutions as the provision of ART continues to expand. Socio-cultural barriers affecting adherence included stigma, HIV misconceptions, HIV serostatus nondisclosure, and limited partner involvement. Collaborations with NGOs to plan media campaigns, community outreach, and education sessions are needed to build community support for WLWH from a culturally sensitive viewpoint and influence community perceptions of HIV and PMTCT, promote disclosure, and enhance healthcare-seeking attitudes.

Previous research has addressed challenges to PMTCT uptake in rural regions (Peltzer et al., 2009, 2011). Results suggested that clinics in rural areas often had limited access to maternal healthcare materials; additionally, communities and patients had limited HIV knowledge. While peer educators, a major theme in previous studies (Peltzer et al., 2011; Sam-Agudu et al., 2015)*,* were not examined in this study, there is continuing need for community and partner education. Patient support groups were viewed favorably by patients, and encouraging attendance could help foster connections and facilitate discussions among women utilizing PMTCT. However, CHCs often lacked PMTCT counseling, training, and experienced space limitations (Ladur et al., 2015). In addition, clinic staff also reported delayed test results, supply/staff shortage, inadequate health record maintenance, and patient oversaturation. These barriers reduced the PMTCT protocol’s effectiveness and affected clinic attendance. Ongoing responses to staff shortages could improve patient outreach, adherence and record keeping, wait-times and clinic attendance. Though recruitment of additional staff is imperative, it should only be undertaken when frequent, comprehensive training in the PMTCT protocol can be guaranteed.

Many of these barriers could be addressed by diverting monetary and physical – resources to rural clinics, which requires external sources and internal motivation to improve the implementation climate. For example, increasing clinic spaces can ease patient flow and ensure privacy, reducing anxiety related to disclosure. Additionally, decreased patient density can reduce exposure to communicable diseases among clinic patients. Small, on-site laboratories and increased testing supply availability could prevent delays in receipt of results and ensure timely follow-up. Although some staff reported borrowing lab supplies, this was not a permanent solution; underlying structural and systemic issues must be addressed. Data management systems such as EMR, while costly, are an acceptable system that would greatly reduce the impact of patient migration on loss to follow-up and curb record entry errors. Such an approach can potentially enhance patient retention and re-engagement.

Finally, previous research has indicated that healthcare workers are motivated by incentives (The CDI Study Group, 2010), and management could apply this strategy in context of PMTCT, as staff often felt unappreciated. Likewise, poor attendance to support groups, misunderstanding of pregnancy among patients, community misconceptions and societal beliefs were reported as barriers to patient adherence. To promote early ANC attendance, patient and community PMTCT education must be ongoing and include early identification of pregnancy, responding to the special needs of women during pregnancy, including education on HIV prevention. Increasing knowledge about HIV could reduce stigma, which could improve societal perceptions of PMTCT protocols. Increasing self-efficacy, assessing readiness to take up PMTCT strategies among both staff and patients may help improve the implementation of PMTCT interventions (CFIR, 2014), as personal attributes, e.g. provider empathy, have been associated with greater patient satisfaction, disclosure, and adherence (Orner et al., 2008).

Men’s involvement in ANC was identified as a clinic- and patient-related challenge and has been cited in previous literature (Ladur et al., 2015; Peltzer et al., 2011). Men’s involvement may require improved engagement strategies and training individuals within the organization to act as champions to overcome societal resistance of male participation in pregnancy. Men in the clinic were sometimes perceived as unconventional and even unacceptable and long waiting periods at the clinic often conflicted with working schedules. Limited clinic space prevented clinic staff from accommodating male partners’ participation, as it reduced privacy or created discomfort for other clinic-goers. Having flexible clinic schedules and using appointment logs may promote better management of clinics’ patient loads and may potentially increase patient satisfaction and male involvement. Though some clinics scheduled one day out of the week to see pregnant women, such schedules may be impractical for working men and women. Scheduling non-working patients during traditional working hours, and working patients or non-working women with working male partners later in the day or on the weekend may reduce time conflicts, staff shortage and space limitations. These suggestions validate previous research on strategies to improve PMTCT, including those emphasizing the importance of male partner involvement (Tsague et al., 2010) and increased clinic staffing (Peltzer et al., 2009, 2011).

### Limitations

The use of an integrated workshop, with staff from upper and lower-level positions participating in the same groups, was used to facilitate cross-discipline problem solving. Due to the perceived lack of support among lower-level staff, participants may not have shared their own solutions as fully as they might have among more restricted groups. Despite these limitations, every effort was made to include multiple perspectives, both in groups and in individual interviews, to minimize these issues. Though many of the challenges and solutions identified in this study have been explored or suggested in previous research (Ladur et al., 2015; Peltzer et al., 2009, 2011; Sam-Agudu et al., 2015), many have solutions suggested in this study have not been implemented. Novel strategies evaluating stakeholder-suggested solutions are needed to address the complex, unique, and multifaceted challenges faced by rural areas with regard to PMTCT protocol implementation and uptake. This study was novel in that it utilized multilevel data collection and analysis by involving individuals at all levels of PMTCT protocol implementation. Furthermore, a validated set of constructs for evaluating the effectiveness of scientific application and implementation, the CFIR (CFIR, 2014), was used to examine challenges and potential solutions to improve PMTCT protocol implementation and uptake, which had previously not been applied to the rural South African context.

## Conclusion

This study examined challenges to PMTCT care in rural SA and identified solutions to improve perception of PMTCT, patient adherence, staff knowledge, and protocol uptake. Results process provided valuable insight into locally appropriate solutions to barriers to PMTCT implementation. Reducing rates of HIV MTCT has been successful in many areas of SA; PMTCT strategies are widely accepted and have resulted in a 52% decrease in MTCT of HIV between 2001 and 2012 (UNAIDS, 2013). Addressing challenges and gaps limiting PMTCT uptake is crucial to increase coverage of the program in rural, low-resource areas. To this end, key stakeholders can play valuable roles in the effort to reduce MTCT of HIV and achieve an AIDS-free generation.

## ORCID

*Violeta J. Rodriguez* http://orcid.org/0000-0001-8543-2061

*Karl Peltzer* http://orcid.org/0000-0002-5980-0876

*Gladys Matseke* http://orcid.org/0000-0002-3002-0286

*Shandir Ramlagan* http://orcid.org/0000-0001-9906-4474

*Sibusiso Sifunda* http://orcid.org/0000-0002-8766-7240

*Guillermo “Willy” Prado* http://orcid.org/0000-0002-9164-4244

*Viviana Horigian* http://orcid.org/0000-0002-3578-0014

*Stephen M. Weiss* http://orcid.org/0000-0001-6266-6148

*Deborah L. Jones* http://orcid.org/0000-0002-4106-8891
